# Supplementary material for: Multi‐dataset identification of innovative feature genes and molecular mechanisms in keratoconus
Source: J Cell Mol Med. 2024 Sep 19;28(18):e70079. doi: 10.1111/jcmm.70079 (PMC11412914; doi:10.1111/jcmm.70079)
Supplement: Supplementary file 2 — Material S2. [file JCMM-28-e70079-s002.zip › 7.GSEA analysis/1.ARL11/KEGG_METABOLISM_OF_XENOBIOTICS_BY_CYTOCHROME_P450.html]

Details for gene set KEGG\_METABOLISM\_OF\_XENOBIOTICS\_BY\_CYTOCHROME\_P450[GSEA]

|  || Dataset | ARL11.group.cls#h\_versus\_l |
| Phenotype | group.cls#h\_versus\_l |
| Upregulated in class | l |
| GeneSet | KEGG\_METABOLISM\_OF\_XENOBIOTICS\_BY\_CYTOCHROME\_P450 |
| Enrichment Score (ES) | -0.3517893 |
| Normalized Enrichment Score (NES) | -1.1173444 |
| Nominal p-value | 0.2813765 |
| FDR q-value | 1.0 |
| FWER p-Value | 0.994 |
Table: GSEA Results Summary

  

Fig 1: Enrichment plot: KEGG\_METABOLISM\_OF\_XENOBIOTICS\_BY\_CYTOCHROME\_P450      
 Profile of the Running ES Score & Positions of GeneSet Members on the Rank Ordered List

  

| SYMBOL | TITLE | RANK IN GENE LIST | RANK METRIC SCORE | RUNNING ES | CORE ENRICHMENT || 1 | UGT2B7 | na | 1178 | 0.368 | 0.0097 | No |
| 2 | GSTA3 | na | 2651 | 0.258 | -0.0021 | No |
| 3 | UGT2B15 | na | 2914 | 0.244 | 0.0194 | No |
| 4 | UGT2B11 | na | 2923 | 0.244 | 0.0482 | No |
| 5 | CYP3A7 | na | 3477 | 0.220 | 0.0584 | No |
| 6 | UGT2B4 | na | 3576 | 0.216 | 0.0813 | No |
| 7 | ADH4 | na | 3617 | 0.215 | 0.1056 | No |
| 8 | UGT1A8 | na | 3762 | 0.209 | 0.1263 | No |
| 9 | CYP2C19 | na | 4519 | 0.182 | 0.1261 | No |
| 10 | UGT1A6 | na | 4803 | 0.172 | 0.1384 | No |
| 11 | GSTA2 | na | 5278 | 0.158 | 0.1435 | No |
| 12 | UGT2B28 | na | 5683 | 0.146 | 0.1491 | No |
| 13 | CYP1A2 | na | 5834 | 0.141 | 0.1616 | No |
| 14 | ADH6 | na | 6053 | 0.136 | 0.1715 | No |
| 15 | CYP3A5 | na | 7636 | 0.099 | 0.1376 | No |
| 16 | CYP2E1 | na | 8693 | 0.079 | 0.1166 | No |
| 17 | DHDH | na | 9329 | 0.070 | 0.1065 | No |
| 18 | UGT2B10 | na | 9893 | 0.062 | 0.0976 | No |
| 19 | MGST2 | na | 10508 | 0.054 | 0.0863 | No |
| 20 | GSTT2 | na | 11478 | 0.042 | 0.0633 | No |
| 21 | ALDH3B1 | na | 11840 | 0.038 | 0.0574 | No |
| 22 | MGST3 | na | 12526 | 0.031 | 0.0413 | No |
| 23 | CYP2B6 | na | 12739 | 0.029 | 0.0387 | No |
| 24 | GSTT1 | na | 13017 | 0.027 | 0.0339 | No |
| 25 | UGT2A3 | na | 13634 | 0.022 | 0.0187 | No |
| 26 | ALDH3B2 | na | 13954 | 0.019 | 0.0118 | No |
| 27 | CYP3A4 | na | 14527 | 0.015 | -0.0030 | No |
| 28 | ADH1B | na | 16426 | 0.001 | -0.0576 | No |
| 29 | GSTP1 | na | 17007 | -0.003 | -0.0740 | No |
| 30 | GSTO1 | na | 17080 | -0.004 | -0.0756 | No |
| 31 | GSTO2 | na | 17085 | -0.004 | -0.0753 | No |
| 32 | CYP2C9 | na | 17344 | -0.006 | -0.0821 | No |
| 33 | GSTK1 | na | 18167 | -0.012 | -0.1044 | No |
| 34 | ADH7 | na | 18569 | -0.014 | -0.1143 | No |
| 35 | CYP2C8 | na | 18873 | -0.016 | -0.1211 | No |
| 36 | GSTZ1 | na | 19033 | -0.017 | -0.1237 | No |
| 37 | GSTM5 | na | 20064 | -0.024 | -0.1505 | No |
| 38 | ALDH3A1 | na | 20262 | -0.026 | -0.1532 | No |
| 39 | CYP2C18 | na | 20676 | -0.029 | -0.1616 | No |
| 40 | UGT2A1 | na | 20689 | -0.029 | -0.1586 | No |
| 41 | GSTM3 | na | 21920 | -0.039 | -0.1894 | No |
| 42 | GSTM2 | na | 23101 | -0.051 | -0.2174 | No |
| 43 | ADH5 | na | 23842 | -0.060 | -0.2316 | No |
| 44 | GSTA4 | na | 25863 | -0.088 | -0.2794 | No |
| 45 | EPHX1 | na | 28375 | -0.140 | -0.3351 | Yes |
| 46 | ALDH1A3 | na | 28596 | -0.146 | -0.3241 | Yes |
| 47 | AKR1C4 | na | 28839 | -0.153 | -0.3129 | Yes |
| 48 | GSTM1 | na | 28996 | -0.156 | -0.2988 | Yes |
| 49 | AKR1C1 | na | 29392 | -0.169 | -0.2902 | Yes |
| 50 | AKR1C3 | na | 30128 | -0.191 | -0.2886 | Yes |
| 51 | CYP2S1 | na | 30431 | -0.202 | -0.2734 | Yes |
| 52 | CYP2F1 | na | 30848 | -0.216 | -0.2597 | Yes |
| 53 | MGST1 | na | 31885 | -0.256 | -0.2592 | Yes |
| 54 | CYP3A43 | na | 32273 | -0.273 | -0.2379 | Yes |
| 55 | GSTM4 | na | 32561 | -0.292 | -0.2115 | Yes |
| 56 | ADH1C | na | 33242 | -0.340 | -0.1908 | Yes |
| 57 | ADH1A | na | 33507 | -0.360 | -0.1556 | Yes |
| 58 | GSTA5 | na | 33671 | -0.375 | -0.1157 | Yes |
| 59 | CYP1A1 | na | 34339 | -0.484 | -0.0774 | Yes |
| 60 | CYP1B1 | na | 34694 | -0.745 | 0.0010 | Yes |
Table: GSEA details [plain text format]

  

Fig 2: KEGG\_METABOLISM\_OF\_XENOBIOTICS\_BY\_CYTOCHROME\_P450      
 Blue-Pink O' Gram in the Space of the Analyzed GeneSet

  

Fig 3: KEGG\_METABOLISM\_OF\_XENOBIOTICS\_BY\_CYTOCHROME\_P450: Random ES distribution      
 Gene set null distribution of ES for **KEGG\_METABOLISM\_OF\_XENOBIOTICS\_BY\_CYTOCHROME\_P450**
